# User preferences for a contraceptive microarray patch in India and Nigeria: Qualitative research on what women want

**DOI:** 10.1371/journal.pone.0216797

**Published:** 2019-06-06

**Authors:** Aurélie Brunie, Rebecca L. Callahan, Christine L. Godwin, Jyoti Bajpai, Funmilola M. OlaOlorun

**Affiliations:** 1 Health Services Research, Washington, DC, United States of America; 2 Contraceptive Technology Innovation, Durham, NC, United States of America; 3 Behavioral, Epidemiological and Clinical Sciences, Durham, NC, United States of America; 4 Independent Consultant, New Delhi, India; 5 College of Medicine, University of Ibadan, Ibadan, Nigeria; USC Keck School of Medicine, Institute for Global Health, UNITED STATES

## Abstract

**Background:**

A new contraceptive microarray patch (MAP) for women is in development. Input on this method from potential end-users early in the product development process is important to guide design decisions. This paper presents the qualitative component of a broader study exploring initial acceptability of the MAP and selected product features. The qualitative research was intended to identify product features that are most salient to end-users and to contextualize preferences around them with subsequent research planned to assess quantitatively the relative importance of those features.

**Methods:**

We conducted 16 focus group discussions and 20 in-depth interviews with women and 20 IDIs with family planning providers in New Delhi, India, and Ibadan, Nigeria. Input from the MAP developer served to identify plausible features of the MAP to include in the focus group discussions and in-depth interviews. Interviews were audio-recorded, transcribed into English, coded, and analyzed to examine key dimensions of MAP features.

**Results:**

Many participants viewed the MAP as potentially easy to use. Interest in self-application after learning correct use was high, especially in India. Participants favored formulations affording protection from pregnancy for three or six months, if not longer. Interest in a shorter-acting MAP was particularly low in Nigeria. Non-desirable MAP features included a potential localized skin rash and pain at application. Views on patch size and location of application were related to the potential for rash and pain, with a desire to permit discreet use and minimize pain. Results indicate that the side effect profile, effectiveness, and pricing are also important factors for acceptability and uptake of a future product.

**Conclusion:**

Study findings indicate that a contraceptive MAP is of potential interest to women and that specific MAP attributes will be important to acceptability.

## Introduction

In 2017, 74% of the 89 million unintended pregnancies in developing regions occurred among women not using contraception, while an additional 10% occurred among women using traditional contraceptive methods [1]. Factors leading to non-use or discontinuation of modern contraceptives include lack of accurate information, cost, access to limited services, and method-related reasons such as concerns about side effects, misperceptions about risk of pregnancy, opposition from family members, or inconvenience [2]. A 2011 analysis of data from Demographic and Health Surveys in Sub-Saharan Africa, South Central Asia, and Southeast Asia found that 71% of women with an unmet need for contraception (i.e. who would like to delay or stop childbearing but are not using a modern contraceptive) reported method-related reasons for not using a modern method [3]. Hence, in addition to improving access to accurate family planning information and to methods, developing new contraceptive products that more adequately meet women’s needs is an integral component of efforts to reduce unmet contraceptive need.

One product in the contraceptive development pipeline is a contraceptive microarray patch (MAP). MAPs have been developed as an alternative to injections that enables delivery of drugs and vaccines using a skin patch that can be self-administered, generates no biohazardous waste and is expected to be inexpensive to manufacture [4, 5]. MAPs contain solid microarrays that penetrate into the upper layers of skin and release coated or encapsulated drug within minutes. Research has led to several published clinical trials and approved cosmetic and medical products [4–7].

Researchers from the Georgia Institute of Technology are developing a novel MAP to serve as a platform for contraceptive delivery for women. Unlike conventional MAPs, which deliver drug as a bolus, the contraceptive MAP is made of biodegradable polymer that slowly releases contraceptive hormone (e.g., levonorgestrel). The microarrays are being designed to rapidly separate from the patch backing so that the patch can be applied for as little as 10 seconds, after which the microarrays remain hidden below the skin’s surface to slowly release contraceptive hormone.

Experience with MAPs in other contexts suggest that a contraceptive MAP could be an attractive contraceptive for use in low- and middle-income countries. For example, a recent phase I clinical trial found that MAPs administering influenza vaccine were a safe, effective, and patient-preferred alternative to hypodermic needles and that they could be painlessly self-administered [8]. In addition, several advantages of MAPs identified in the context of their potential for vaccine delivery in low- and middle-income countries could prove relevant in the contraceptive field, including improved access through administration by lower-level cadres or self-application, simplified storage, distribution, and disposal requirements, reduced risk of sharps, and expected low manufacturing costs [9]. Product development for contraceptive application is still at a pre-clinical stage; two variants of dissolving microarrays are being considered, with additional decisions to be made on specific patch features such as location or duration of application, among others [10]. In addition to technical feasibility, exploring women’s attitudes toward a contraceptive MAP and their perceptions of its characteristics is important to guide design decisions and ensure that future products will fit their needs and the circumstances of their lives.

The research presented in this paper is part of a user preferences study of the contraceptive MAP in India and Nigeria. Using a sequential, exploratory design, this study included qualitative research that informed the design of a discrete choice experiment survey to quantify the relative importance of selected MAP features. This paper describes the first phase of the study: the qualitative research. The objectives of the qualitative research were to explore initial acceptability of the MAP, identify product features salient to end-users, and provide context on preferences.

## Materials and methods

### Study setting

The qualitative research was conducted in New Delhi, India, and Ibadan, Nigeria, two populous countries with different contraceptive mixes. Specific sites within the two countries were selected based on convenience. Modern contraceptive prevalence among married women in India is 47.8%, with female sterilization being the most commonly used method [11]. In Nigeria, 10% of married women and 54% of unmarried women use modern contraception [12]. Short-acting methods are most common in this setting, including injectables among married women and male condoms among unmarried women. Unmet need is 12.9% among married women in India, and 16.1% among married women and 21.8% among unmarried women in Nigeria [11, 12].

### Eligibility and sampling

The cross-sectional, exploratory qualitative research included focus group discussions (FGDs) and in-depth interviews (IDIs) with women, and IDIs with family planning providers. Eligible women in India were aged 15–49 and either married or in union. In Nigeria, eligible women were sexually active (in the last 30 days) and aged 18–49, irrespective of marital status, or aged 15–17 and married. Providers were eligible if they provided modern methods beyond just male condoms and/or female sterilization.

We recruited a purposive sample of women in each country, stratified by ever/never use of modern contraception, and, in Nigeria only, by marital status to account for possible diversity in opinions among these groups. Because of the high prevalence of female sterilization in India, sterilized women in this setting were categorized as ever/never user based on contraceptive use prior to being sterilized. The sample also allowed representation of both peri-urban and urban contexts. Indian women were mobilized by clinic-based and outreach staff from one peri-urban and one urban reproductive health clinic. In Nigeria, women were recruited through primary health care facilities in one urban and one peri-urban local government area with the assistance of the clinic staff; some women were also directly recruited from communities near the clinics. Providers in both countries were purposively selected to represent the public and private sectors and achieve variation in designation. Following evidence that 80% saturation can be achieved within three FGDs, we aimed to complete four FGDs per stratum, when aggregated [13]. Since IDIs were intended to complement FGDs, we estimated that 5 IDIs per aggregated stratum should be sufficient [14].

### Data collection and ethical considerations

This qualitative research was conducted between July-August 2017 in India and December 2017-March 2018 in Nigeria. In each country, three local, masters-level consultants were each assigned one type of interview (FGDs or IDIs with women, IDIs with provider); each of the FGDs also included one other consultant as a note taker. All FGDs and IDIs were conducted in either English or the local language (Hindi or Yoruba), depending on participants’ preference. Three topic guides were developed with similar content but tailored to each type of interview. Each interview began with a description of the MAP (S1 Fig), and participants were shown prototype patches and given the opportunity to handle them. Prototypes purchased from Micron Biomedical were needle- and drug-free, and made a click sound when depressed onto the skin.

Development of topic guides was informed by discussions with the lead product developer at Georgia Institute of Technology to identify an initial set of relevant MAP attributes and plausible levels for each (Table 1). Some of the attribute levels included in the guides were modified between the two countries based on additional discussion with the developer after reviewing preliminary results from India. For example, based on input from the developer, all potential MAP application locations included in topic guides were chosen to be flat and lean areas of the body favorable to patch adherence and drug delivery. We removed shoulder blade from the list of potential locations for Nigeria due to impracticality for self-use and added kneecap and foot to get input on additional locations. Size options were also adjusted to reflect more likely options based on evolving technical considerations provided by the developer.

**Table 1:** Attributes and levels included in topic guides.

Attributes	Levels included in India	Levels included in Nigeria
**Administration**	Provider-administered Self-administered	Provider-administered Self-administered
**Duration of protection**	1 week 1 month 3 months 6 months	1 week 1 month 3 months 6 months
**Location of application**	Top of wrist Shoulder blade	Top of wrist Kneecap Top of foot
**Number of patches per application**	One Two	One Two
**Pain at application**	Finger prick Maximum acceptable pain	Pin prick Maximum acceptable pain
**Size (round shape)**	0.75-inch diameter 1-inch diameter 1.25-inch diameter (surface area shown as rectangle)	1-inch diameter 1.25-inch diameter 1.5-inch diameter
**Skin reaction**	Rash for few hours Rash for few days Rash for one week or longer	Rash for few hours Rash for few days Rash for one week or longer
**Wear time**	On and off Maximum acceptable wear time	On and off Maximum acceptable wear time

The guides included an exploration of initial thoughts on the method, and a list of broad and follow-up questions organized around various possible MAP attributes and levels. Participants were also asked how much they would be willing to pay for an MAP. In FGDs, research assistants recorded the range of acceptable prices across participants, and the midpoint was used in summarizing results across interviews. The guides also allowed for a discussion of additional levels for the proposed attributes beyond those originally specified and an exploration of other possible relevant attributes.

All FGDs and IDIs with women were conducted in private rooms at health facilities, and providers were interviewed at their place of practice. All interviews were audio-recorded, then transcribed and translated into English for analysis. Written consent was obtained from all participants. All participants were compensated for their travel and time; FGD participants were also offered refreshments. Sigma Institutional Review Board in India, the Oyo State Research Ethical Review Committee in Nigeria, and FHI 360’s Protection of Human Subjects Committee in the United States approved this study.

### Data analysis

Transcripts were uploaded into NVivo 11 for coding and analysis. For each country, transcripts were divided between two analysts and coded using a pre-established scheme combining structural and thematic codes related to the attributes included in the guide and to perceptions of the MAP [15]. To assess the reliability of coding, both analysts coded and compared results for 10% of India transcripts, with additional periodic checks for Nigeria. Three team members prepared analysis memos examining the key dimensions of each code separately for each country. This step was focused on extracting all attributes and levels (initially included in the guide or emerging from the interviews) and examining participants’ reactions to these attributes and levels, as well as to the MAP more broadly. We used matrices in Excel to summarize results and examine possible differences by country, participant type, or participant characteristics.

## Results

A total of 54 women in India and 60 women in Nigeria participated in the FGDs and IDIs (Table 2). All providers but one community pharmacist in Nigeria were women. Providers had experience in their current designation ranging from 1–44 years in India and 3–33 years in Nigeria. The mean durations of FGDs and IDIs with women were 109 and 74 minutes, respectively, in India, and 104 and 82 minutes in Nigeria. IDIs with providers lasted, on average, 45 minutes in India and 60 minutes in Nigeria.

**Table 2:** Sample size and participant characteristics.

Type of data collection	Total number of data collection events	Total number of participants	Mean age (range), years	Mean parity
**INDIA**				
FGDs with women	• 4 with married, ever users • 4 with married, never users	44	28 (18–43)	1.7
IDIs with women	• 5 with married, ever users • 5 with unmarried, never users	10	28 (19–43)	1.0
IDIs with providers	• 4 with public sector providers • 6 with private sector providers	10	50 (26–72)	—
**NIGERIA**				
FGDs with women	• 2 with married, ever users • 2 with unmarried, ever users • 2 with married, never users • 2 with unmarried, never users	50	32 (18–49)	1.9
IDIs with women	• 2 with married, ever users • 3 with unmarried, ever users • 3 with married, never users • 2 with unmarried, never users	10	31 (19–48)	1.4
IDIs with providers	• 5 with public sector providers • 5 with private sector providers	10	45 (29–62)	—

### Patch administration

Many women, especially in India, as well as half of Indian providers and all but one provider in Nigeria felt that the MAP was easy to apply based on the explanations they received and their viewing and handling of the prototype patches. They said the application was simple, fast, and did not require any instruments. Another advantage reported by several women and providers in both countries is adherence because the MAP does not require daily intake. Several women noted that applying the MAP was preferable to having to swallow drugs, receiving an injection, or undergoing an invasive procedure. Several Indian women liked that the MAP was not designed to “go inside the body” like the copper IUD, and a few ever users in an FGD and a couple of providers in Nigeria liked that it did not require exposing “private parts.”

*See this method has no detailed procedures*, *no instrument is to be used*, *nor has it to be used daily*, *so I think these are all benefits*. *It is easily applicable and approachable*. *(public sector provider*, *India IDI—IU_02*)

When asked about their views on self- and provider-administration, more Indian women expressed interest in self-use than not in both FGDs and IDIs. In Nigerian FGDs, provider-administration was preferred overall, but most IDI participants favored self-application. Among 41 Indian women and 43 Nigerian women who provided a response, 33 women in India and 18 in Nigeria found self-use appealing. However, one-third of those in India and half of those in Nigeria went on to say that the MAP should be administered by a provider the first time, and several women also proposed that an instruction should be made available when the MAP is launched in the market. All providers wanted women to receive the MAP from a provider: most Indian providers thought this was important for the first time, whereas most Nigerian providers felt women should only receive the MAP from providers.

Perceived advantages of self-use discussed by women primarily included convenience but also discretion. In India, women reported challenges related to clinic hours interfering with other activities and to cultural norms making it difficult for women to leave their homes alone. In Nigeria, several women talked about saving time and not missing work, and of a general dislike for going to the clinic when not ill.

Yet FGDs and IDIs also highlight some concerns around self-use. Many women and providers mentioned possible issues or lack of confidence around applying the MAP correctly, for example with the right amount of pressure or in the right location. A few women liked the “click” sound the prototype patch made when depressed on the skin because they took it as a sign that the MAP had been sufficiently pressed onto the skin. A number of women and some providers had specific reservations about self-use related to women fearing microarrays and the potential for pain.

*I can go to a doctor [to have it applied] as mostly women get scared when they see needles*. *If a needle would prick*, *I know that I will take it off immediately*. *This might harm the body or [it] might make the method ineffective*. *But as against this*, *if you go to a doctor*, *then they would know how to put the method properly*. *(32-year-old*, *married never user with one child*, *India FGD—INMF2_2*)

Some women and providers in both countries talked about possible shortcomings of self-use in the broader context of care, including lack of proper screening for contraindications and of appropriate counseling at initiation.

*[The health workers] are the ones who know how you react and how things are going*, *so they will guide you*, *they will tell you this is how to do it*, *this is the advantages and disadvantages…first*, *before you take it*, *you have to seek the doctor’s advice*, *after that you can be doing it your own self at home*. *(40-year-old*, *married woman with three children*, *current user of male condoms*, *Nigeria IDI–NEI_2*)

### Pain at application

Many women and providers talked about apprehension around needles, which they associated with pain. A few participants even advised against using the term “needles” either as part of the product’s name or in describing it.

*In the description it is mentioned that the method contains a lot of needles*. *So rather than saying that we can also say that the method contains a lot of medicine*. *And the medicine dissolves in the body*. *Women are scared of needles*. *So*, *if you tell them that this method contains a lot of needles*, *they will get scared*. *(24-year-old*, *married never user with three children*, *India FGD–INMF1_6*)

Some Indian women anticipated that applying the MAP would be more painful than an injection because multiple needles, and not just one, were involved. In India, a few women and providers had concerns related to needles entering and dissolving in the body, including whether they may accumulate through repeated MAP use, could get “stuck and hurt inside”, or may be visible.

Participants in the vast majority of data collection events said that pain akin to an ant bite (India), pin prick (Nigeria), or injection (both countries) would be acceptable, and only a few participants said that there should be less pain than with an injection or no pain. A few women and providers said the needles in the MAP were on par or preferable to the pain from other methods like injectables or the copper IUD. Several Nigerian women also noted that the benefits of preventing pregnancy offset fear of pain.

*Anyone that says they want to do something should already have it at the back of their mind that there is a trade-off*. *So*, *I do not think that little thing*, *that little pain*, *is too much*. *It is not much at all for us to have what we desire*, *what we want to plan our home*. *(34-year-old*, *married never user with no children*, *Nigeria FGD–NNMF1_1*)

Many participants were concerned about pain duration. Many women, especially in Nigeria, indicated that pain should only be instantaneous at the time of application; there should not be residual pain.

### Location of application

In each country, participants were prompted about their reactions to different places on the body where the MAP could be applied (wrist and shoulder blade in India; wrist, kneecap, and foot in Nigeria). In general, participants did not strongly support any of these locations. Perspectives on wrist application in both countries and on application on the shoulder blade in India were mixed. There was more opposition than support among Nigerian women for applying the MAP on the kneecap, and even more so for the foot. In Nigeria, several women and a couple of providers commented on the proposed locations all being “bony.”

Women and providers were also asked to suggest additional locations. In both countries, the most popular locations were the upper arm and the thigh, followed by the buttocks in Nigeria or the broader midsection (waist, hips, and buttocks) in India. A few women who were suggesting the upper arm or the buttocks said these locations were normal and familiar for injections.

In both countries, the main consideration was whether the location of application could make the skin reaction visible to others. Although not all explicitly referred to the skin reaction, participants in most data collection events expressed a preference for locations that could be hidden under clothing. The wrist was generally considered to be exposed, but perspectives on whether other locations were hidden varied. For example, several Indian women said that the shoulder blade was covered by clothing, but a few noted it would be exposed with a deep-neck dress. A few participants had contrasting views on whether open or hidden locations were best for healing of a possible localized skin rash because of such factors as contact with air or water or rubbing with clothes. At the same time, some women wanted the MAP to be applied in a location visible to them to enable them to monitor the skin reaction.

Many women, mostly Indian (perhaps because of the shoulder blade option), also reflected on whether specific locations would be compatible with self-application.

*I think it can also be applied on the thighs as I can see it while applying*, *and yet that part is not visible to others…I can see for the reddish reaction and if it leads to some kind of skin problem*, *then I will get to know about it*. *(43-year old*, *married IUD user with two children*, *India FGD—IEMF4_1*)

Another factor for several women in both countries was differential pain tolerance across different areas of the body, leading them to anticipate pain or complications around joints and/or favoring fleshier parts. Many women also considered how residual pain or complications at certain locations may adversely impact their ability to perform certain activities, especially in Nigeria (although this may have been influenced by the locations being discussed). This included cooking or manual work (wrist), kneeling for prayer or walking (kneecap), or wearing shoes or walking (foot).

In several FGDs and IDIs with women in India, women connected the location of application to the potential effectiveness of the MAP. Examples of their arguments include thicker skin holding the method better or, conversely, preventing the needles from going inside; and perceptions of increased effectiveness if the MAP is applied to the midsection of the body closer to reproductive organs.

When asked whether a unique location should be specified for the MAP or if multiple locations should be possible, participants’ responses were split, although slightly more supported having some flexibility. Proponents of a single location advocated for a unique site proven to guarantee product effectiveness. Those supporting multiple locations said that giving women the choice of their preferred location would increase acceptability and also allow users to rotate the site of administration. Some of them, however, felt that choices should be limited to a few pre-selected locations.

### Patch size

Assuming a round shape, three patch sizes were discussed in each country (sizes refer to the diameter): two were similar (1 in and 1.25 in); the third option in India was smaller than the two common patches (0.75 in), while in Nigeria it was larger (1.5 in). Smaller size options (0.75 in and 1 in in India and 1 in and 1.25 in in Nigeria) were preferred by both women and providers in each country, with participants typically considering the largest size shown to them to be too big (1.25 in in India and 1.5 in in Nigeria).

One of the main perceived advantages of smaller sizes was more discretion, especially in India, largely due to the smaller surface area that may be marked by a rash and a better ability to conceal a smaller patch print under clothing. Relatedly, among some participants, there was a perception that smaller patches could fit on more parts of the body, whereas larger sizes would be more limited in where on the body they could be applied. Several women also indicated that smaller patches afforded more discretion in terms of storage or portability.

In addition, a number of women in both countries said that applying patches with a larger size would be more painful due to a greater number of needles underneath the patch.

*[The] smaller*, *the better*. *It will leave a smaller mark*. *That is why this is better*. *There will be less pain with this*. *So*, *this smaller size is better*. *(36-year-old*, *married never user with two children*, *India IDI—IE_I09*)

A few women said that ensuring effectiveness was essential, and that any of the proposed sizes would be acceptable if it allowed the MAP to work effectively. A few women and providers in India were doubtful that the smallest size would contain enough hormones to be effective, while a few women were wondering how size would affect the duration of protection afforded by each option. A few participants also had the perception that women may require different patch sizes depending on their body weight.

### Wear time

In general, participants did not appear to be overly concerned about wear time. When prompted about this scenario, many women liked the idea of being able to apply and immediately peel off the MAP. However, several women in both countries and most Indian providers were not comfortable with instant removal, primarily because they were not sure that the wear time would be sufficient for the MAP to be effective.

*I am thinking that they should leave it for some few minutes*, *maybe like two minutes*, *and that will even make it look as if you are administering a drug to people*. *So*, *we should leave it on the skin a little*. *Maybe it is one minute or two minutes before removing it*. *(26-year old*, *single woman with no children currently using male condoms*, *Nigeria FGD–NEUF1_6*)

Some women in both countries said they would want to wear the MAP less than one minute, but almost three times as many women were willing to keep it on for a maximum duration of one to five minutes, with many Indian women and some Nigerian women prepared to apply it even longer. A number of women and a few providers said the wear time should be long enough to guarantee effectiveness, whereas a few women in each country factored in pain tolerance in explaining why wear time should not be too long.

### Duration of protection

Among the duration options being discussed, a longer duration of three or six months was largely preferred over one week or one month, with participants in most data collection events also spontaneously expressing interest in longer durations ranging between one and five years. There was very limited support for a patch that would last one week in India and none in Nigeria, and perspectives on a one-month patch were mixed in both countries.

Some women and providers linked longer durations to a greater willingness to tolerate pain at application, and a few participants also indicated that the duration of protection should exceed that of any potential skin reaction from applying the MAP.

Some women and providers referred to the three-month injectable and, in India, the copper IUD as benchmarks for justifying preference for longer durations. A few Nigerian providers specifically indicated that a six-month formulation could help meet the demand for a longer-acting injectable they were observing among their clients.

*According to my research on our people and they are asking if it is possible to get an injectable that will provide contraceptive cover for six months*? *An injectable for one year*? *So*, *let’s say [the MAP] can work for six months or for a year*. *You know people prefer this injectable…they’ve known of a month*, *they’ve known of two months*, *likewise three months*, *they are seeking for six months*. *(public sector provider*, *Nigeria IDI—NU3*)

With shorter-acting options, a number of Nigerian women and providers in both countries expressed concern about keeping track of time and remembering to re-apply the method and, often envisioning a scenario requiring visits to the health center in their responses, about the burden of frequent visits to a clinic considering work and family responsibilities.

In Nigeria, several never users and providers also held the view that the duration of protection should be aligned with birth spacing intentions and that a short-acting method would not afford a sufficient spacing interval.

*What I can say about that one-month issue is that the minimum should not be less than a year*. *Someone who wants to use family planning actually wants to put space between her children*. *She can’t just say “I am ready” after three months…*. *It is that of one year*, *two years*, *three years*, *that is called family planning*. *But ah*, *ah*! *Doing family planning within one*, *three months*, *and thereafter say that she is not doing family planning again*, *that is not a plan at all*. *(35-year-old*, *married never user with three children*, *Nigeria FGD–NNMF2_3*)

On the other hand, some Nigerian women said that the duration of use should not be too long to accommodate changes in fertility intentions. One Nigerian never user and some providers in both countries also talked about the advantage of a shorter duration to allow for discontinuation as a strategy for managing side effects. Either in relation to pregnancy intentions or side effect management, several participants did not like that the effects of the MAP could not be reversed. Some women and providers, especially in Nigeria, said that different MAP products with different duration of protection should be developed so as to allow women to choose a patch of their preferred duration.

### Skin reaction at application

In general, participants did not like the idea of the MAP causing any kind of skin reaction upon application. In 31 of 36 data collection events with women and 14 of 20 with providers, participants said that a localized rash would be noticeable by others and raise questions.

Many women and a few providers were concerned about pain or itching associated with a rash. Especially in India, a number of women and a few providers were also worried that the rash could progress into an infection.

When asked about acceptability of several possible rash durations, participants in almost all data collection events in India and in most data collection events in Nigeria indicated that a rash that would wear off after a few hours would be acceptable. Perspectives on longer rash durations were more divided. While more women said they would tolerate a rash that would last a few days than those who said they would not, women in most data collection events were not accepting of the idea of a rash that would extend to a week or longer, especially in Nigeria.

Whereas some women and several Nigerian providers said they would prefer a mild rash lasting a few days to a stronger reaction lasting only a few hours, participants in 30 of 36 data collection events with women and nine of 20 IDIs with providers were willing to trade off intensity for a shorter duration. Although only a limited number of participants expanded on their rationale, Indian women’s concerns over infection appeared to increase with duration rather than intensity of skin reaction, while they associated increased intensity to greater pain. In both countries, some women also said that longer-lasting or more visible rashes could both attract increased attention by others.

*[If the rash lasts for one week]*, *people will ask questions*. *And in fact*, *if it lasts for a week*, *even I will start wondering if the mark has got infected*. *(26-year old*, *married women with two children using pills and male condoms*, *India FGD–IEMF8_3*)

### Number of patches at application

Interviews explored the acceptability of applying two patches simultaneously to extend the duration of protection. Of 71 women and 12 providers who provided a response, 61 women and three-quarters of providers favored applying only one patch at a time. Among those that were concerned about applying multiple patches simultaneously, several participants said it could increase pain or cause a larger rash, some thought the higher combined dose of hormones could heighten side effects, and a few doubted the efficacy of this technique in affording protection from pregnancy over a longer period of time.

*If you say they will give two, that one can be too much for the body because it is hormonal*, *because maybe you [are] supposed to give 1mL and you give 2 mL, the reaction will be too much. I mean the side effect will be too much, it will be too much. (public sector provider, Nigeria IDI—NU_2)*

### Method packaging and stocking

Participants were also asked if they would be interested in procuring several patches at the same time in the context of self-use. Overall, participants in slightly more data collection events preferred getting multiple patches at once, whereas participants in more data collection events in Nigeria preferred getting patches one at a time. Perceived advantages of acquiring multiple patches included increased convenience from reduced visits and confidence about continuity of supply that may otherwise be jeopardized in case of shortages in the market. A few women also saw some financial advantages to this approach, including the ability to align purchases with cash flow or the possibility of savings from buying bulk.

Conversely, participants identified challenges with stocking patches at home, including expiry dates, affordability, and misplacing products. Several married women and a couple of providers also mentioned the risk of accidental disclosure if others, including primarily husbands or children, were to find MAPs around the home, although a few Nigerian women felt that the MAP was small enough to hide easily.

In addition, a few Nigerian women wondered whether storage requirements were compatible with stocking the product at home. Most providers in Nigeria were opposed to giving multiple MAPs to women to keep at home because of concerns over sharing of product with others, exceeding dosage, using expired products, or losing the patches.

### Side effect profile

Side effects were not mentioned in the initial description of the product. However, at least 30 of the 36 data collection events with women and 19 of the 20 provider IDIs encompassed some spontaneous discussion of side effects, inclusive in particular of bleeding changes and weight changes, in the context of using the MAP. Several women in India excitedly assumed that the MAP had no such side effects since these were not mentioned by research assistants when introducing the method. For the most part, participants inquired about the side effect profile, expressed general concerns about side effects, or said they hoped side effects would be minimal. In Nigeria, a number of women were also concerned about the MAP causing unacceptable delays in return to fertility.

*What bothers me is that a known devil is better than an unknown one, we do not yet know about this thing that is being developed. Now the pills that we are using, injection, there are some that react*, *maybe someone who receives it may not menstruate, there may be someone who receives it and will feel she has family planning in her body system but will still get pregnant…I have to see few people who have done it and report that there are no problems associated with it before I can do it. (19-year old, single never user, Nigeria IDI—NNI_4)*

### Pricing

In India, women appeared to be willing to pay between 5 and 300 rupees for a one-month product (median price of 50 rupees or US $0.72), and 20 and 500 rupees for a three-month product (median price of 125 rupees or US $1.81). As a comparison point, private providers reported a price of 25–75 rupees (US $0.36–1.08) for pills, approximately 200 rupees (US $2.89) for three-month injectables, and 500 rupees (US $7.23) for the copper IUD.

In Nigeria, private providers offering these methods reported a price of 30–400 naira (US $0.08–1.11) for pills, 100–500 naira (US $0.28–1.39) for injectables, and 200–850 naira (US $0.55–2.35) for implants. Although methods are free in the public sector, some providers reported charging about 100 naira (US $0.28) for injectables and 200 naira (US $0.55) for implants. Women reported they would pay 50–5000 naira for a one-month MAP (median price of 275 naira or US $0.76) and 150–15,000 naira for a three-month MAP (median price of 550 naira or US $1.52).

Whether in absolute terms or relative to other methods, IDIs consistently underscore that the price of the MAP should not be too high. Many women and providers, especially in Nigeria, said that the method should be affordable across income groups, including for poor women. Some data collection events with women and providers highlighted price as a key consideration for choosing the MAP over other contraceptive options, or for contraceptive uptake. In addition, a number of participants reported that other methods were either free or cheap, and used this to justify why the MAP should be priced along similar lines.

*You see those injectable, they are free if you get to the [health] centers…Aside from the fact that you just give those in charge of it two hundred or three hundred naira*, *it is free. They will even pack condoms…They can say it is money for the gloves that they will use to pick it. It is free. So, the way it is, I cannot pay more than five hundred naira for three months. (28-year old, single woman with no children currently using male condoms, Nigeria FGD–NEUF1_1)*

A few participants in each country suggested initially introducing the MAP for free or at very low price to attract users, and then increase the price.

*It should be less, maybe a marginal difference is there*, *but it should be slightly less than the cost of [an] injection…because we can increase the cost later also, once people start using this method…But if we keep the cost higher at first, then people will have problem in using this method. People will not use it, no matter how many advantages there are for this method (25-year old, married never user with no children, India IDI—IN_I07)*

Some competitive advantages of the MAP were discussed that may justify a slightly higher price than for pills or injections, including that the MAP does not require daily intake, offers potential for self-use (India), and was perceived as less painful or intimidating than an injection (Nigeria). Several participants also indicated that a reduced side effect profile compared to other methods would be a clear differentiating factor, with a few Nigerian women further noting that guaranteed effectiveness was a key factor in justifying price.

## Discussion

This paper reports on the qualitative research conducted to examine initial acceptability of a contraceptive MAP in India and Nigeria and explore the perspectives of end-users on its possible different features. Our approach was based on iterative discussions with the product developer and qualitative data collection with women and family planning providers in the two countries. In addition to the learnings presented here, this research lays the foundation for the design of a discrete choice experiment survey that will permit quantifying women’s preferences for MAP attributes and the trade-offs they are willing to make between them.

Our findings do provide important insight into the overall potential acceptability of a contraceptive MAP and an early indication of user preferences related to its design. Overall, women and providers reacted positively to the idea of a contraceptive MAP, finding the product to be non-invasive, simple and fast to apply, and easy to use.

Self-use is regarded as an important possible advantage of the MAP by developers and donors. Recent research in the United States reports similar efficiency of investigator- and self-administration of placebo dissolving MAPs, with most participants feeling at least somewhat confident that they had applied the patch correctly [16]. Our findings confirm the potential acceptability of the MAP in two countries for self-application after teaching women how to properly apply the patch and offering appropriate counseling and reassurance about pain and needles at method initiation. They also point to some features that could help increase confidence in proper method use, such as a click sound confirming the method has been sufficiently and appropriately pressed. As shown by recent research on Sayana Press, self-use is a welcome development in overcoming some of the barriers to contraceptive continuation [17].

At the current stage of development, developers are still investigating the technical feasibility of various durations of protection against pregnancy. To keep the scope of inquiry broad, this exploratory qualitative research considered a range of possible durations, including a six-month option. Participants strongly preferred durations at least equivalent to the three-month injectable, but our results indicate demand for even longer-acting products. Though the MAP may be unlikely to protect from pregnancy for more than six months given the difficulty of incorporating larger doses into MAPs, this finding is consistent with data from the Performance Monitoring & Accountability 2020 (PMA2020) surveys in seven states of Nigeria showing a slight increase in use of long-acting methods [18] and with the history of high use of long-acting and permanent methods in India. In terms of product development, the strong interest in long-acting options among participants makes progestin-only formulations potentially relevant to the design of the MAP in addition to combined hormonal formulations. Given frequent concerns about the side effect profile of the product, implications of these different formulations, especially for menstrual bleeding, also need to be kept in mind.

Findings also point to some less desirable features of a MAP that may affect its ultimate acceptability and uptake including skin reaction and pain at application. In addition to intrinsic preferences for not feeling pain or incurring a rash, we found that participants actively referred to trade-offs between these and other MAP attributes. Moreover, these perspectives were inscribed in a broader context through considerations such as the potential for discreet use or access. Concerns over erythema were cosmetic and related to disclosure of contraceptive use. Discussions around potential skin rash were closely related to concerns about location of application and patch size since these attributes would jointly dictate potential for discreet use. Participants also worried about pain associated with a localized rash and the potential for infection, particularly as rash duration increases. Plausible levels of pain at application appear to stay within acceptable levels; however, participants were also concerned about residual pain, which developers do not expect to occur. Moreover, experience with MAPs to date have not reported pain or infection at MAP application sites, and skin reactions have been limited to mild, localized, transient erythema [4–7].

Participants were not enthusiastic about any of the locations pre-identified for proper MAP application through consultations with the product developer, i.e., flatter and leaner areas that would facilitate proper separation of microarrays and optimal drug delivery. Women want fleshy, hidden locations to minimize pain and maximize discretion. Patch size, intensity of any possible skin reaction, and the potential for residual pain and how it may interfere with regular activities may be critical factors in reaching a compromise between technical feasibility and user acceptability.

Method effectiveness emerged as a cross-cutting theme related to concerns about applying proper pressure, appropriate locations for application for proper drug delivery, and sufficient wear time for penetration of hormones. Despite the difference in contexts, women in both countries appeared to be willing to pay somewhat comparable amounts for a contraceptive MAP–approximately 75 cents for a one-month product and about twice this amount for a three-month product. Our estimates are crude, however, and further research is needed on this topic. Nonetheless, our findings indicate that along with MAP effectiveness, ensuring overall affordability and a minimal price difference with other contraceptive options will be important components of successful product development and introduction strategies.

While many findings were similar across both countries, interesting differences also emerged. For example, providers and to some extent women in Nigeria were less quick to endorse self-application than their Indian counterparts, partly due to concerns over improper use. Perhaps relatedly, women in the two countries had contrasting preferences for stocking patches, with more interest in procuring multiple patches at once and stocking them at home in India. Although participants in both countries expressed more interest in three- or six-month formulations of the MAP, interest in shorter durations like one week or one month appeared to be particularly low in Nigeria.

### Limitations

Firstly, while qualitative findings provide valuable information, they will be augmented by findings from the subsequent discrete choice experiment survey to examine the extent to which they may be generalized and support more specific recommendations. Secondly, while we tried to recruit participants with different backgrounds, for practical reasons, the study was conducted in urban and peri-urban settings in the two countries and women and providers in more rural settings may have different perspectives. Thirdly, framing the description of the MAP to give sufficient background on its different features while not overwhelming participants and maintaining flexibility to allow subsequent, sequential exploration of different level options for several attributes is a challenge. Possible biases may result from the order in which each attribute was introduced, and anchoring effect may also have occurred when reacting to proposed attribute levels. For example, perspectives on wear time and patch size may have been influenced by expectations set up during the interview based on the levels being discussed. In addition, because of possible conceptual interactions between product attributes, not fixing the level of other attributes when discussing level options for any given attribute complicated both discussions and analysis. The fact that side effects were not included in the initial description also misled some participants to think a contraceptive MAP had little or no side effects, which may have affected some of the responses related to other attributes or overall acceptability of the MAP. We used different level options for some attributes between the two countries, which makes comparison difficult. In India, but not Nigeria, we also used a picture to illustrate the skin reaction that likely overstated the intensity of the possible rash and could therefore have amplified concerns. And finally, while it provides an initial ballpark, the measure of willingness to pay used in this study is crude since the target product profile was neither fixed nor fully specified. The ability to gain further insight into potential pricing of the MAP relative to other methods is limited by differences in probing, with some interviews asking about factors that may lead women to choose the MAP over other contraceptive options like the pills or injections and others asking explicitly about willingness to pay more for the MAP than for other methods. In addition, women were not always aware of the price of other contraceptive methods to use as a benchmark.

## Conclusion

This exploratory qualitative research provides evidence of initial acceptability of a contraceptive MAP and its different possible features. It offers initial empirical guidance on target product characteristics that will be supplemented by findings from a larger quantitative survey using discrete choice methodology.

## Supporting information

S1 Fig Description of the contraceptive microarray patch. (DOCX)
