# Supplementary material for: User preferences for a contraceptive microarray patch in India and Nigeria: Qualitative research on what women want
Source: PLoS One. 2019 Jun 6;14(6):e0216797. doi: 10.1371/journal.pone.0216797 (PMC6553846; doi:10.1371/journal.pone.0216797)
Supplement: S1 Fig — (DOCX) [file pone.0216797.s001.docx]

# Supporting information

## S1. Description of the contraceptive microarray patch.

Now I am going to show you a new type of contraceptive that is currently being developed. This method is not available yet. We will share your thoughts about this new method with the scientists who are developing it.

This method is a patch that is pressed onto the skin and has very small needles underneath it. The needles are made of a material that dissolves to release a product into the body. The product is like the one in existing contraceptives like pills and the injection. The method will hurt very slightly or not at all when it is put on the skin. The method is put on the skin for a short time and then is removed. It keeps protecting against pregnancy for a longer period of time after it is removed. The method will be safe and highly effective at preventing pregnancy.

This is an example of what the method might look like, but without needles. The method that will be developed may look different from this. It may be a different size or shape. And here is a picture of what the needles look like under a microscope. You can imagine that there will be lots of these tiny needles on the underside of the patch.
